# From Deep-Sea Natural Product to Optimized Therapeutics: Computational Design of Marizomib Analogs

**DOI:** 10.3390/ijms262412159

**Published:** 2025-12-18

**Authors:** Nasser Alotaiq, Doni Dermawan

**Affiliations:** 1 Health Sciences Research Center (HSRC), Imam Mohammad Ibn Saud Islamic University (IMSIU), Riyadh 13317, Saudi Arabia; 2 Department of Applied Biotechnology, Faculty of Chemistry, Warsaw University of Technology, 00-661 Warsaw, Poland; doni.dermawan.stud@pw.edu.pl

**Keywords:** 20S proteasome β5 subunit, frontier molecular orbital, glioblastoma, marizomib, molecular dynamics, pharmacophore

## Abstract

The proteasome β5 subunit plays a central role in protein degradation and is an established therapeutic target in glioblastoma. Marizomib (MZB), a natural β5 inhibitor, has shown promising anticancer activity, yet suboptimal pharmacological properties limit its clinical translation. Using a comprehensive computational approach, this study aimed to identify and characterize novel MZB analogs with improved binding affinity, stability, and drug-like profiles. An integrative in silico study was performed, including molecular docking, frontier molecular orbital (FMO) analysis, pharmacophore modeling, molecular dynamics (MD) simulations over 200 ns, MM/PBSA binding free energy calculations, and per-residue energy decomposition. ADMET profiling evaluated the pharmacokinetic and safety properties of MZB and top-performing analogs. Docking and pharmacophore modeling revealed strong complementarity between MZB analogs and the β5 catalytic pocket. MD simulations showed that MZBMOD-77 and MZBMOD-79 exhibited exceptional structural stability with low RMSD values (0.40–0.42 nm), persistent binding within the active site cavity, and significant disruption of hydrogen-bond networks in the active loop regions Ala19–Lys33 and Val87–Gly98. MM/PBSA analysis confirmed their superior binding free energies (−19.99 and −18.79 kcal/mol, respectively), surpassing native MZB (−6.26 kcal/mol). Per-residue decomposition highlighted strong contributions from Arg19, Ala20, Lys33, and Ala50. ADMET predictions indicated improved oral absorption, reduced toxicity, and favorable pharmacokinetics compared to native MZB. This integrative computational study identifies MZBMOD-77 and MZBMOD-79 as promising next-generation proteasome β5 inhibitors. These analogs mimic and enhance the inhibitory mechanism of native MZB, offering potential candidates for further optimization and preclinical development in glioblastoma therapy.

## 1. Introduction

Glioblastoma (GBM) remains the most aggressive and lethal primary brain tumor in adults, with a median overall survival of only 15–18 months despite maximal surgical resection followed by radiotherapy and temozolomide chemotherapy [1,2]. The poor prognosis is attributed to high tumor heterogeneity, intrinsic therapy resistance, and the limited ability of many systemic agents to cross the blood–brain barrier (BBB) [3,4]. Consequently, there is a critical need to identify and optimize small molecules that can effectively penetrate the central nervous system (CNS) while targeting essential oncogenic pathways [5,6]. The ubiquitin–proteasome system (UPS) represents one such attractive therapeutic target. By regulating protein degradation, the proteasome plays a central role in controlling cell cycle progression, apoptosis, and stress responses [7,8,9]. Inhibition of the proteasome has revolutionized the treatment of multiple myeloma, where agents such as bortezomib and carfilzomib significantly improved patient outcomes [10,11]. However, first- and second-generation proteasome inhibitors suffer from poor BBB penetration and dose-limiting toxicities, restricting their clinical utility in brain tumors [12].

Marizomib (salinosporamide A) is a marine-derived, irreversible, pan-proteasome inhibitor that distinguishes itself from its predecessors by its ability to efficiently cross the BBB [13,14]. Structurally, Marizomib (MZB) contains a unique γ-lactam–β-lactone pharmacophore that covalently modifies the proteasome β subunits, particularly β5 (chymotrypsin-like), while also inhibiting β1 (caspase-like) and β2 (trypsin-like) sites [15,16]. Preclinical studies demonstrated robust anti-tumor activity in glioblastoma models, providing the rationale for clinical translation [17]. Unfortunately, in a large randomized phase III trial (EORTC 1709/CE.8), the addition of MZB to standard temozolomide-based chemoradiation failed to improve overall survival compared to standard of care and was associated with higher rates of grade 3/4 toxicities [18]. These findings highlight a central paradox: although MZB possesses favorable pharmacological properties, including BBB penetration, its clinical efficacy in GBM is limited by systemic toxicity and insufficient therapeutic selectivity. Rational structural modification of MZB offers a promising strategy to overcome these limitations. Specifically, fine-tuning its electrophilic reactivity, improving β5-subunit selectivity, and reducing off-target interactions could yield analogs with improved therapeutic indices. Recent advances in computational drug design, including molecular docking, pharmacophore modeling, molecular dynamics (MD) simulations, and in silico ADMET prediction, provide a cost-effective and systematic approach to explore such analogs before committing to resource-intensive synthesis and biological testing [19,20,21].

The present study aims to employ an integrative computational pipeline to design, evaluate, and prioritize novel MZB analogs. Using molecular docking against the human 20S proteasome β5 subunit, pharmacophore modeling, highest occupied molecular orbital-lowest unoccupied molecular orbital (HOMO–LUMO) analysis, long-timescale MD simulations, molecular mechanics/Poisson-Boltzmann surface area (MM/PBSA) free energy estimation, and in silico absorption, distribution, metabolism, excretion, and toxicity (ADMET) screening, we seek to identify modifications that optimize efficacy while reducing toxicity. By integrating structural biology with modern cheminformatics, this work addresses the translational gap in proteasome inhibitor development for CNS malignancies and may provide a blueprint for the next generation of targeted proteasome therapeutics. We hypothesize that rational structural modifications of MZB can yield novel analogs with enhanced proteasome selectivity, reduced systemic toxicity, and retained BBB penetration, thereby improving its therapeutic potential for glioblastoma treatment.

## 2. Results

### 2.1. Structure Alignment and Similarity Analysis

To assess the structural space of designed MZB analogs, a structure alignment and similarity analysis was performed, focusing on representative modifications categorized into halogenation, alkyl/aryl substitutions, hydroxyl/ether modifications, lactone/lactam ring variations, ester modifications, and fused or polycyclic transformations. The similarity between each analog and native MZB was quantified using the Tanimoto coefficient (FP2), with values closer to 1.0 denoting higher structural resemblance. As shown in Table 1, the analogs display a broad range of similarity scores (0.446–0.927), reflecting the diversity of chemical modifications introduced to the MZB scaffold. Halogenation emerged as a relatively conservative strategy, with analogs such as MZBMOD-1 (0.927) and MZBMOD-4 (0.909) retaining high similarity to MZB. These substitutions involved the incorporation of bromine or chlorine atoms, which preserved the core cyclic scaffold while subtly altering electronic properties and steric bulk. Such modifications are often leveraged to enhance pharmacokinetic stability or fine-tune lipophilicity without drastically perturbing biological recognition motifs. The relatively high similarity values suggest halogenated derivatives may conserve binding conformations comparable to the parent compound.

In contrast, alkyl and aryl substitutions produced more pronounced structural deviations, with similarity scores ranging from moderate (0.847 for MZBMOD-25) to low (0.601 for MZBMOD-22). This reduction can be attributed to introducing bulky aromatic moieties or flexible alkyl side chains extending beyond the compact MZB lactone-lactam framework. While such substitutions decrease overall scaffold similarity, they provide opportunities to explore new binding interactions, potentially accommodating different subpockets of the proteasome or enhancing solubility profiles. For instance, aryl-linked sulfonyl groups (MZBMOD-25) may introduce additional hydrogen bonding capacity, whereas smaller alkyl ethers (MZBMOD-22) could improve membrane permeability at the expense of structural conservation. Hydroxyl and ether modifications demonstrated intermediate similarity scores (0.721–0.900), reflecting the relatively subtle impact of polar functional group replacements within the cyclic backbone. For example, MZBMOD-36 (0.900) retains much of the native scaffold, with the addition of a cyclic ether moiety providing local polarity adjustments that could influence solubility and hydrogen-bonding interactions. Conversely, MZBMOD-38 (0.721) introduces more substantial modifications to side-chain polarity, which, while reducing overall similarity, could alter binding specificity or reduce off-target effects. This highlights the delicate balance between structural resemblance and the introduction of chemical diversity necessary for analog optimization.

Lactone and lactam ring modifications (MZBMOD-50 and MZBMOD-56) displayed moderate similarity scores (0.565–0.626). Such alterations directly impact the rigid cyclic architecture central to MZB’s bioactivity, leading to significant scaffold rearrangements. Although these modifications reduce structural resemblance, they can expand chemical diversity by probing alternative ring topologies. Importantly, the introduction of heteroatoms within the cyclic system may affect hydrogen bonding, metabolic stability, and conformational rigidity, making these analogs valuable for assessing structure–activity relationship (SAR) boundaries. Ester-modified analogs, such as MZBMOD-68 (0.449) and MZBMOD-72 (0.446), exhibited the lowest similarity scores among all categories. This drastic reduction indicates a significant departure from the MZB core framework, as ester substitutions tend to increase molecular flexibility while disrupting the native lactone-lactam backbone. Although these modifications risk diminishing proteasome-targeting fidelity, they may enhance hydrolytic susceptibility, potentially yielding prodrug-like properties with improved pharmacokinetics. Their role, therefore, may lie less in direct proteasome inhibition and more in exploring alternative chemical scaffolds inspired by MZB. Finally, fused and polycyclic analogs (MZBMOD-77 and MZBMOD-79) demonstrated similarity scores in the mid-range (0.542–0.626). These analogs represent significant cyclization and scaffold reorganization events, leading to complex 3D architectures. Such modifications can impart improved rigidity and conformational constraint, which are often favorable for high-affinity binding, albeit at the cost of lower similarity to the native compound. The balance between rigidity and steric bulk introduced by fused systems warrants further computational docking and dynamic simulations to evaluate their bioactivity potential.

**Figure 1:**
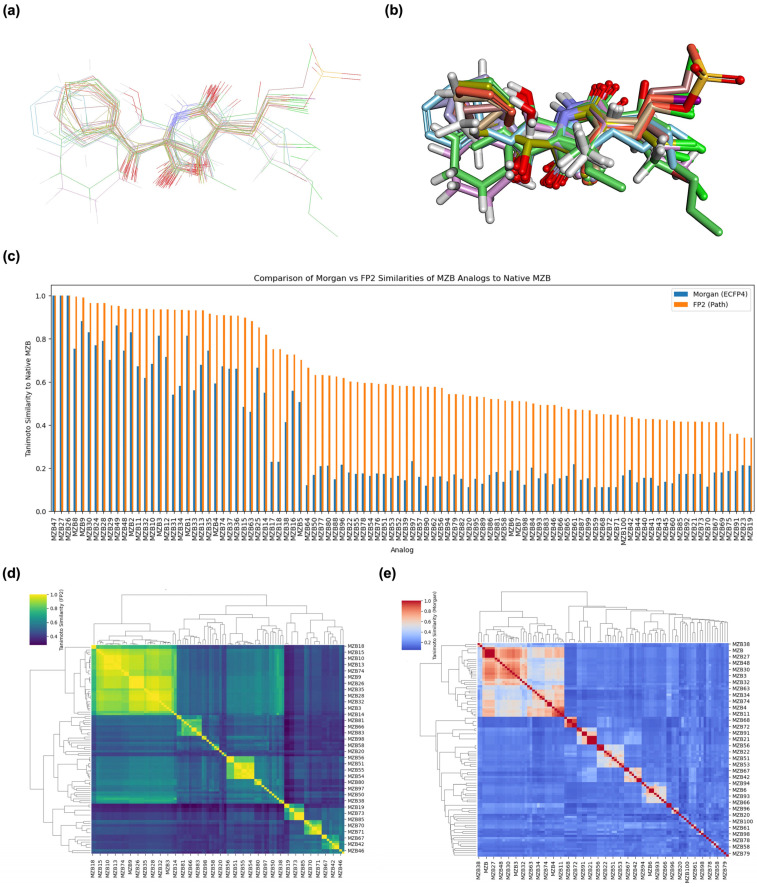
Structural alignment and similarity analysis of Marizomib (MZB) and its 100 designed analogs. (**a**) 2D alignment of MZB with representative analogs, illustrating preservation of the central bicyclic core while allowing structural variation at peripheral substituents. (**b**) 3D conformational superposition of MZB and analogs, showing consistent alignment of the pharmacophoric core with divergent substituent orientations. (**c**) Comparison of Tanimoto similarity scores of each analog to native MZB using two molecular fingerprinting methods: FP2 (path-based, orange) and Morgan (ECFP4, blue), highlighting differences in sensitivity to local versus global structural changes. (**d**) Hierarchical clustering heatmap (FP2-based) of pairwise analog similarities, where yellow regions indicate high similarity and dark blue regions indicate lower similarity. (**e**) Hierarchical clustering heatmap (Morgan ECFP4-based) of pairwise analog similarities, where red regions denote high similarity clusters and blue regions reflect lower similarity relationships.

The structural diversity of MZB analogs was first examined through alignment overlays to assess the extent of scaffold conservation across the designed library. As illustrated in Figure 1a, the analogs generally maintain the core β-lactone-γ-lactam framework of native MZB, with deviations localized primarily to substituent positions on the cyclic backbone. The superimposition highlights both conserved rigid regions, likely essential for proteasome binding, and flexible regions where chemical modifications were introduced. A more detailed 3D alignment (Figure 1b) demonstrates that despite variation in side-chain orientation and steric bulk, most analogs adopt conformations compatible with the MZB pharmacophore, suggesting that modifications successfully explored chemical diversity without entirely disrupting the bioactive scaffold. Quantitative similarity analysis using Tanimoto coefficients further supported these observations. As shown in Figure 1c, FP2 path-based fingerprints consistently yielded higher similarity scores than Morgan circular fingerprints, reflecting FP2’s sensitivity to conserved atom paths within the cyclic scaffold. Several halogenated and hydroxyl/ether-substituted analogs displayed similarity values exceeding 0.85, indicating close resemblance to native MZB. In contrast, analogs incorporating ester substitutions or more complex fused-ring systems showed reduced similarity (<0.50), consistent with their structural departure from the native backbone. This divergence underscores the ability of the analog library to span a broad structural landscape, from conservative derivatives to scaffold-innovative chemotypes.

Hierarchical clustering of the analogs revealed distinct similarity-driven groupings. FP2-based clustering (Figure 1d) highlighted several tightly clustered analog families, particularly among halogenated and hydroxylated derivatives, which indicates localized chemical changes around the conserved core. Conversely, Morgan-based clustering (Figure 1e) produced more diffuse groupings, better capturing global scaffold rearrangements such as lactone-to-lactam substitutions and polycyclic modifications. Interestingly, ester-modified analogs consistently segregated into isolated clusters, reflecting their pronounced departure from the MZB pharmacophore. These clustering results suggest that different fingerprinting approaches capture complementary aspects of chemical similarity: FP2 excels at identifying minor local modifications, whereas Morgan fingerprints are more sensitive to broader scaffold rearrangements. The combined structural alignment, similarity scoring, and clustering analyses reveal that the designed MZB analog library balances scaffold preservation with chemical innovation. High-similarity analogs (e.g., halogenated, hydroxyl/ether derivatives) are likely to conserve the essential proteasome-binding interactions of native MZB while offering opportunities for fine-tuning physicochemical properties such as lipophilicity and solubility. In contrast, lower-similarity analogs (e.g., ester and fused-ring modifications) may represent more radical scaffold explorations, with the potential to uncover novel binding modes or proteasome subunit selectivity.

### 2.2. Molecular Docking Results, Binding Pose, and Binding Affinity Analysis

Molecular docking was employed to evaluate the binding potential of MZB, BA (a known proteasome activator), and a panel of top-performing analogs with the PSMB5 catalytic subunit of the 20S proteasome, the primary target of this study. As summarized in Table 2, native MZB demonstrated a binding energy of −7.13 kcal/mol with a HADDOCK score of −27.1 ± 0.4 a.u., establishing a benchmark for comparison. Its binding was stabilized by multiple hydrogen bonds with residues Arg19, Thr21, Gly23, and Gly47, residues previously reported to contribute to proteasome inhibition [22]. In contrast, BA exhibited a slightly weaker binding energy (−6.98 kcal/mol) and fewer stabilizing contacts, forming only a single hydrogen bond with Gly47, consistent with its role as an activator rather than an inhibitor. Among the analogs, several compounds exhibited stronger binding affinities than native MZB. Notably, MZBMOD-77 achieved the most favorable docking profile, with a binding energy of −8.09 kcal/mol and a HADDOCK score of −34.8 ± 0.4 a.u. Similarly, MZBMOD-79 and MZBMOD-50 also demonstrated improved energetics (−7.83 and −7.37 kcal/mol, respectively), with HADDOCK scores below −33 a.u. These analogs consistently reproduced the critical hydrogen bonding pattern observed in native MZB, particularly involving Arg19, Thr21, Gly23, and Gly47. The conservation of these interactions underscores their importance in anchoring ligands to the PSMB5 active site and supports the hypothesis that hydrogen bonding similarity is a reliable criterion for analog prioritization.

Energetic decomposition further highlighted differences between analogs. For MZBMOD-77 and MZBMOD-79, van der Waals and electrostatic interactions contributed substantially to the enhanced binding, suggesting that their fused or polycyclic modifications allowed improved steric complementarity and charge stabilization within the active pocket. Interestingly, MZBMOD-93 displayed a strong electrostatic contribution (−42.2 kcal/mol) but a higher RMSD (1.5 Å), suggesting a less stable or alternative binding orientation that may reduce its reliability as a lead scaffold. Conversely, MZBMOD-99, despite showing comparable binding energy (−7.30 kcal/mol), relied heavily on van der Waals contacts with limited electrostatic stabilization and fewer hydrogen bonds, likely explaining its moderate performance relative to other top analogs. The comparison of binding poses revealed that the top analogs maintained the essential hydrogen bonding network and introduced favorable steric contacts that improved desolvation energies and docking stability. For instance, the lactone/lactam-modified MZBMOD-50 formed an additional hydrogen bond with Thr1, suggesting potential opportunities for enhancing selectivity through interactions outside the conserved MZB pocket. Meanwhile, fused-ring analogs such as MZBMOD-77 and MZBMOD-79 appeared to exploit extended hydrophobic surfaces within PSMB5, possibly conferring higher potency but also raising questions about off-target interactions that warranted further selectivity screening. To address this, supplementary docking was performed against the β1 (PSMB6) and β2 (PSMB7) subunits of the proteasome. Native MZB bound less strongly to these off-targets (−6.76 and −6.54 kcal/mol, respectively) compared to β5. By contrast, MZBMOD-77 and MZBMOD-79 exhibited improved binding across all three subunits: PSMB6 (−7.98 and −7.74 kcal/mol, respectively) and PSMB7 (−7.85 and −7.32 kcal/mol, respectively). While these values remain weaker than their β5 affinities, the noticeable improvement relative to MZB suggests that off-target engagement cannot be excluded and warrants further experimental evaluation. Importantly, the ∆∆G values confirmed that both analogs maintained preferential binding for β5 over β1/β2, consistent with their intended inhibitory selectivity.

**Figure 2:**
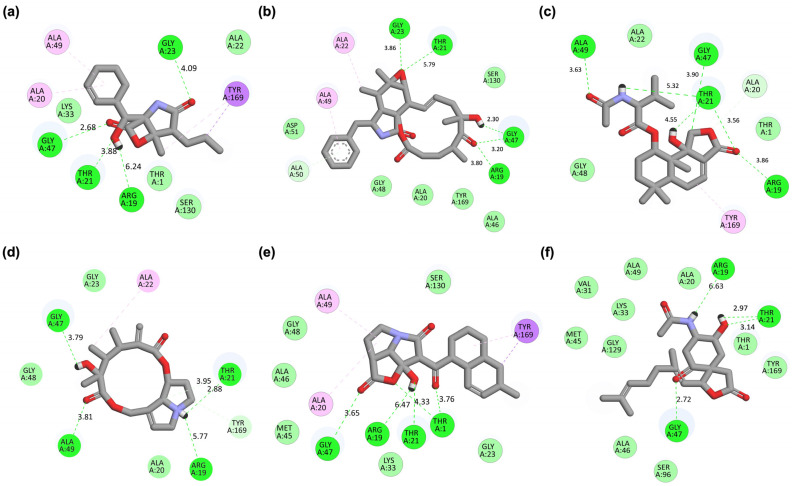
Two-dimensional interaction maps of Marizomib (MZB) and its best-performing analogs with the PSMB5 binding site. (**a**) PSMB5_MZB complex. (**b**) PSMB5_MZBMOD-77 complex. (**c**) PSMB5_ MZBMOD-79 complex. (**d**) PSMB5_MZBMOD-93 complex. (**e**) PSMB5_MZBMOD-50 complex. (**f**) PSMB5_ MZBMOD-99 complex. The interaction types are color-coded as follows: hydrogen bonds (bright green), carbon-hydrogen bonds (light green), van der Waals interactions (pale green), Pi-Alkyl (pink), and Pi-Sigma (purple).

To further elucidate the binding mechanisms underlying the docking results, 2D interaction maps were generated for MZB and its best-performing analogs with the PSMB5 active site (Figure 2). Native MZB (Figure 2a) established a robust hydrogen-bonding network with Arg19, Thr21, Gly23, and Gly47, supported by additional van der Waals and π-interactions with Ala20, Ala49, and Tyr169. This interaction pattern is consistent with previously reported structural studies, reinforcing the role of these residues in stabilizing proteasome inhibitors within the catalytic pocket. Multiple hydrogen bonds near the catalytic threonine (Thr1) and Arg19 suggest strong anchoring of the β-lactone moiety, a critical pharmacophore element of MZB. Among the analogs, MZBMOD-77 (Figure 2b) maintained hydrogen bonds with Arg19, Thr21, Gly23, and Gly47, mirroring the native binding mode. However, its fused-ring modification allowed additional van der Waals contacts with surrounding Ala residues, suggesting an enhanced fit into the hydrophobic sub-pocket. This complementarity may explain the improved binding energy observed in docking, positioning MZBMOD-77 as a particularly promising candidate. Similarly, MZBMOD-79 (Figure 2c) exhibited an extensive hydrogen bond network involving Arg19, Thr21, and Gly47, with supplementary van der Waals stabilization. Interestingly, the analog displayed a π-interaction with Tyr169, a residue near the hydrophobic pocket, which may further strengthen ligand stabilization.

MZBMOD-93 (Figure 2d) also reproduced key hydrogen bonds with Gly23, Thr21, Arg19, and Gly47, but in a more rigid ring conformation. This rigidity restricted van der Waals complementarity and increased RMSD values in docking, suggesting a less flexible pose despite retaining essential contacts. By contrast, MZBMOD-50 (Figure 2e) displayed a distinctive interaction profile. In addition to maintaining bonds with Arg19, Thr21, and Gly47, it engaged with Thr1 through hydrogen bonding. This additional interaction could enhance binding stability or modulate inhibitory potency by more directly targeting the proteolytic mechanism of PSMB5. Lastly, MZBMOD-99 (Figure 2f) retained some canonical hydrogen bonds (Arg19, Thr21, Gly47) but with fewer stabilizing van der Waals contacts and a higher dependence on π-stacking with Tyr169. While the hydrogen bond conservation supports its classification as a top-performing analog, the reduced van der Waals contribution aligns with its slightly weaker docking profile compared to MZBMOD-77 or MZBMOD-79.

**Figure 3:**
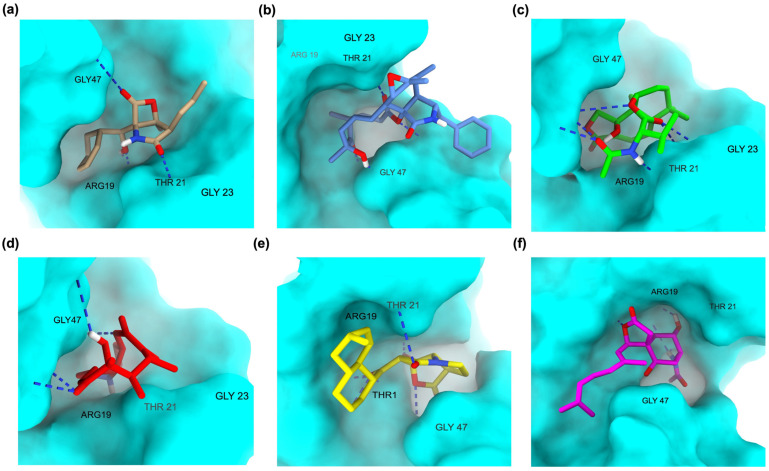
Comparative 3D binding poses of Marizomib (MZB) and its best-performing analogs in the PSMB5 ligand-binding domain (LBD). (**a**) PSMB5_MZB complex. (**b**) PSMB5_MZBMOD-77 complex. (**c**) PSMB5_ MZBMOD-79 complex. (**d**) PSMB5_MZBMOD-93 complex. (**e**) PSMB5_MZBMOD-50 complex. (**f**) PSMB5_ MZBMOD-99 complex.

The binding orientation of native MZB within the PSMB5 catalytic pocket reveals a compact fit, with the β-lactone core positioned deep inside the binding groove and the phenyl moiety extending outward toward the solvent region (Figure 3a). This arrangement enables stable anchoring of the pharmacophoric core while allowing peripheral groups to orient flexibly, ensuring both hydrogen bond stabilization and favorable van der Waals interactions. Analog MZBMOD-77 displayed a pose closely resembling native MZB but with a more extended projection of its aromatic substituent into the adjacent hydrophobic cavity (Figure 3b). This orientation not only conserved the anchoring geometry but also enhanced the filling of unoccupied sub-pockets, which likely accounts for its stronger docking score. MZBMOD-79 adopted a slightly rotated orientation relative to MZB, where the fused ring system tilted deeper into the binding groove (Figure 3c). This shift allowed tighter packing within the hydrophobic cleft while maintaining the exact directional alignment of the β-lactone core. The conformational flexibility observed in this analog suggests a favorable entropic contribution to binding.

A distinct pattern was seen in MZBMOD-93, which assumed a more rigid, circular orientation within the cavity (Figure 3d). The macrocyclic structure limited rotational freedom, keeping the ligand fixed in a perpendicular pose compared to MZB. While this rigidity enforces a snug fit, it may reduce adaptability within the pocket, potentially lowering binding efficiency relative to more flexible analogs. MZBMOD-50 demonstrated the most unique orientation, positioning its extended moiety toward the catalytic channel where Thr1 is located (Figure 3e). This alignment suggests a closer interaction with the catalytic machinery, which may enhance inhibitory potency through direct interference with proteolytic activity. The analog’s orientation appears optimized for deeper engagement rather than lateral surface interactions. In contrast, MZBMOD-99 exhibited an elongated pose stretching across the binding groove (Figure 3f). Although this extension allowed contact with both ends of the pocket, its orientation left part of the hydrophobic cavity underutilized, reducing overall complementarity. This suboptimal positioning may explain its comparatively weaker binding score.

The interaction profiles of MZB, BA, and the top-performing analogs with PSMB5 revealed apparent differences in the extent and diversity of non-covalent contacts formed at the ligand-binding domain (Table 3). Native MZB established a balanced network of carbon–carbon (C–C), carbon–oxygen (C–O), and carbon–nitrogen (C–N) contacts, with moderate contributions from oxygen–oxygen (O–O) and nitrogen–oxygen (N–O) interactions. This pattern indicates a stable but moderately dense interaction interface, consistent with MZB’s established inhibitory potency. By comparison, BA exhibited fewer total contacts across most interaction categories, particularly in nitrogen-involving interactions (N–O, N–N, N–X). The absence of nitrogen-centered contacts suggests weaker hydrogen-bonding capacity and limited polar stabilization compared to MZB. This reduction aligns with BA’s lower docking score and weaker binding affinity, reinforcing that its interaction profile is less optimized for PSMB5 recognition. Among the analogs, MZBMOD-77 exhibited the highest overall interaction counts, with nearly a 1.5-fold increase in C–C, C–O, and C–N interactions compared to native MZB. Importantly, this analog showed elevated O–O and N–O contacts likely contribute to enhanced electrostatic complementarity within the binding site. The dense interaction network of MZBMOD-77 is consistent with its superior docking score, supporting its potential as a structurally optimized derivative.

MZBMOD-79 also demonstrated a substantially enriched interaction profile relative to MZB, although slightly lower than MZBMOD-77. Its elevated O–X and N–O interactions suggest that additional substituents may orient polar groups toward solvent-exposed residues, enhancing solvation and hydrogen-bonding stabilization. Similarly, MZBMOD-50 showed a strong interaction count, particularly in the C–O and N–O categories, consistent with its unique binding orientation near the catalytic Thr1, which promotes polar stabilization. In contrast, MZBMOD-93 displayed interaction counts closer to those of native MZB, suggesting that its more rigid macrocyclic orientation limited opportunities for forming additional stabilizing contacts. Despite achieving slightly higher N–O and N–N interactions, its overall interaction density was relatively modest, which may explain its weaker performance compared to MZBMOD-77 and MZBMOD-79. Finally, MZBMOD-99 demonstrated a broad and rich interaction network, with notably high contributions from nitrogen-centered interactions (N–O, N–N, N–X). This suggests enhanced hydrogen-bonding potential and polar stabilization. However, despite the high contact count, its elongated orientation in the binding groove may prevent optimal complementarity, leading to less efficient energy stabilization compared to the best-performing analogs.

### 2.3. Frontier Molecular Orbital (HOMO–LUMO) Results of Marizomib and Its Analogs

**Figure 4:**
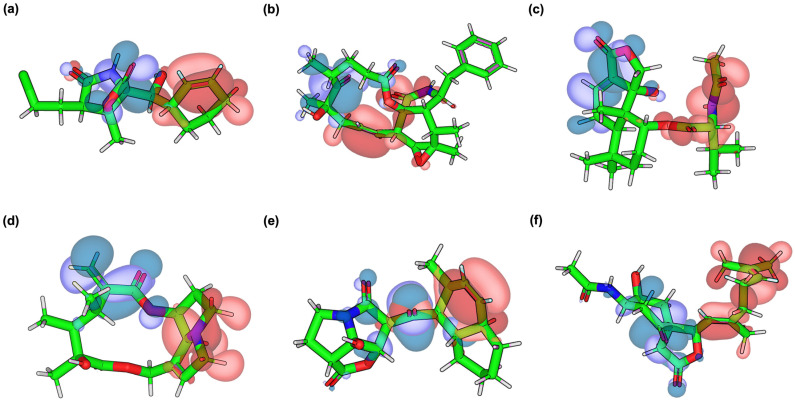
HOMO–LUMO profiles of Marizomib (MZB) and its best-performing analogs. (**a**) MZB. (**b**) MZBMOD-77. (**c**) MZBMOD-79. (**d**) MZBMOD-93. (**e**) MZBMOD-50. (**f**) MZBMOD-99. Blue regions represent HOMO localization, while red regions denote LUMO distribution.

The FMO analysis was conducted to gain insights into the electronic properties and reactivity of MZB and top-performing analogs. As illustrated in Figure 4, the distribution of the HOMO and LUMO varies among the compounds, revealing distinct charge-transfer patterns that may influence binding affinity to PSMB5. The corresponding orbital energies, HOMO–LUMO gaps, and dipole moments are presented in Table 4. In native MZB (Figure 4a), the HOMO density (blue) is localized around the lactam core and neighboring substituents, while the LUMO (red) is primarily distributed across the aromatic terminal. This separation suggests electron transfer from the polar headgroup to the hydrophobic ring system, which may facilitate interactions with both polar and hydrophobic residues within the PSMB5 catalytic pocket. The relatively large HOMO–LUMO gap (5.95 eV) indicates moderate chemical stability, while the higher dipole moment (6.43 D) reflects strong polarity, potentially enhancing solubility and favorable electrostatic complementarity in the binding site.

MZBMOD-77 (Figure 4b) displayed a more extensive HOMO delocalization across the macrocyclic scaffold, while the LUMO remained concentrated near the aromatic substituent. This broader distribution implies enhanced electron-donating potential, increasing the likelihood of stabilizing π–π and hydrogen-bonding interactions. Its reduced energy gap (5.60 eV) suggests slightly greater electronic reactivity compared to MZB, while its significantly lower dipole moment (2.72 D) may reduce solvent polarity-driven interactions but could improve membrane permeability. MZBMOD-79 (Figure 4c) exhibited a pronounced spatial separation, with the HOMO density localized around the macrocyclic moiety and the LUMO confined to the extended substituent. This charge-transfer pattern implies strong adaptability to polarized environments within the proteasome pocket. The compound also showed a relatively narrow energy gap (5.23 eV) and the highest dipole moment among all analogs (8.26 D), suggesting high electronic reactivity combined with enhanced polarity. These features may account for its superior binding profile and strong stabilizing interactions.

By contrast, MZBMOD-93 (Figure 4d) showed more rigid and localized orbital distributions, with HOMO density on one side of the macrocycle and LUMO density on the opposite side. This arrangement limits intramolecular charge delocalization, consistent with its lowest HOMO–LUMO gap (4.92 eV), which indicates high reactivity but potentially reduced stability. The relatively low dipole moment (2.91 D) suggests weaker polarity-driven interactions, which may partly explain why its binding performance was lower compared to MZBMOD-77 and MZBMOD-79 despite favorable reactivity. MZBMOD-50 (Figure 4e) showed an overlap between HOMO and LUMO along the conjugated core, suggesting better internal electronic communication and polarizability. This feature, together with a balanced HOMO–LUMO gap (5.57 eV), indicates stable yet sufficiently reactive behavior. Interestingly, MZBMOD-50 displayed the lowest dipole moment (1.58 D), implying reduced polarity, which could enhance hydrophobic contacts within the PSMB5 pocket. Finally, MZBMOD-99 (Figure 4f) exhibited HOMO confinement on the central scaffold with LUMO distribution at the terminal substituents, mirroring the pattern of MZB but with more pronounced separation. Its moderate HOMO–LUMO gap (5.33 eV) suggests balanced stability and reactivity, while the high dipole moment (7.88 D) points toward strong polarity and favorable electrostatic complementarity.

### 2.4. Pharmacophore Modeling Results

**Figure 5:**
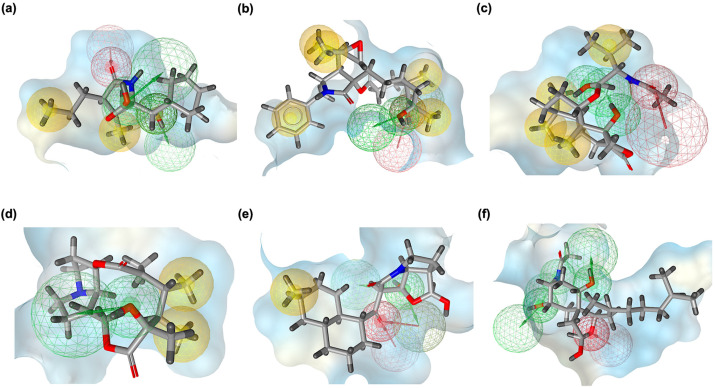
Three-dimensional pharmacophore modeling. (**a**) PSMB5_MZB complex. (**b**) PSMB5_MZBMOD-77 complex. (**c**) PSMB5_MZBMOD-79 complex. (**d**) PSMB5_MZBMOD-93 complex. (**e**) PSMB5_MZBMOD-50 complex. (**f**) PSMB5_MZBMOD-99 complex. Yellow spheres indicate hydrophobic interactions, green arrows represent hydrogen bond donors, and red arrows signify hydrogen bond acceptors.

Pharmacophore modeling was employed to rationalize the docking and FMO findings by identifying the critical interaction features driving recognition between PSMB5 and MZB or its analogs (Figure 5). The resulting pharmacophore maps highlighted the contributions of hydrogen bond donors, hydrogen bond acceptors, and hydrophobic moieties to binding stability, thereby revealing how structural modifications in the analogs reshaped interaction patterns within the catalytic pocket. In the native MZB–PSMB5 complex (Figure 5a), the pharmacophore model revealed a balanced interaction profile, characterized by multiple hydrogen bond donor and acceptor features flanking the lactam moiety, supported by strategically placed hydrophobic contacts. This distribution is consistent with MZB’s dual engagement of polar catalytic residues and hydrophobic sub-pockets, explaining its established inhibitory potency. The dense overlap of donor/acceptor features also aligns with its high dipole moment and polar adaptability observed in FMO analysis.

The MZBMOD-77 complex (Figure 5b) displayed an expanded set of hydrophobic pharmacophore spheres along the extended macrocyclic scaffold, while retaining critical hydrogen bond donor and acceptor features near the binding interface. This shift emphasizes enhanced hydrophobic complementarity without compromising polar contacts. Combined with its broader HOMO delocalization and reduced dipole moment, MZBMOD-77 achieves a favorable balance between lipophilicity and electronic adaptability, rationalizing its superior binding energy compared to native MZB. For MZBMOD-79 (Figure 5c), pharmacophore mapping revealed a striking polarization of features, with hydrogen bond acceptor/donor regions localized near the macrocyclic headgroup, while hydrophobic elements extended toward the distal substituent. This spatial segregation mirrors the FMO-derived HOMO–LUMO separation, highlighting its adaptability to polarized proteasomal environments. The dense clustering of pharmacophoric features, combined with its high dipole moment, likely contributes to its robust hydrogen-bonding and electrostatic complementarity, explaining its superior docking score and binding stability.

In contrast, MZBMOD-93 (Figure 5d) exhibited a pharmacophore map dominated by hydrophobic features, with relatively fewer and spatially restricted hydrogen bond interactions. This rigidity is consistent with its localized orbital distributions and low dipole moment, suggesting limited polar adaptability despite high electronic reactivity. Consequently, while MZBMOD-93 showed theoretical chemical reactivity, its pharmacophore profile indicates suboptimal interaction diversity, which explains its weaker overall binding performance. The MZBMOD-50 complex (Figure 5e) displayed a unique pharmacophore arrangement, where hydrogen bond acceptor/donor features aligned along the conjugated core, co-localizing with several hydrophobic contacts. This overlap suggests strong internal polarizability and flexible adaptation to the PSMB5 pocket, consistent with its balanced HOMO–LUMO gap. However, the minimal dipole moment observed for this analog indicates reduced polarity-driven interactions, pointing instead to its reliance on hydrophobic stabilization near catalytic residues such as Thr1. Finally, the MZBMOD-99 complex (Figure 5f) revealed a dense pharmacophore distribution, particularly enriched in hydrogen bond donor and acceptor sites, alongside peripheral hydrophobic interactions. This feature-rich map mirrors its high dipole moment and extended LUMO distribution, suggesting strong potential for polar complementarity. However, despite its pharmacophoric richness, the docking orientation placed some features sub-optimally relative to key residues, diminishing the efficiency of these interactions. This disconnect explains why MZBMOD-99 formed many stabilizing contacts yet underperformed compared to MZBMOD-77 and MZBMOD-79.

### 2.5. MD Simulations Reveal Structural Stability and Interaction Profiles of Marizomib and Its Analogs

**Figure 6:**
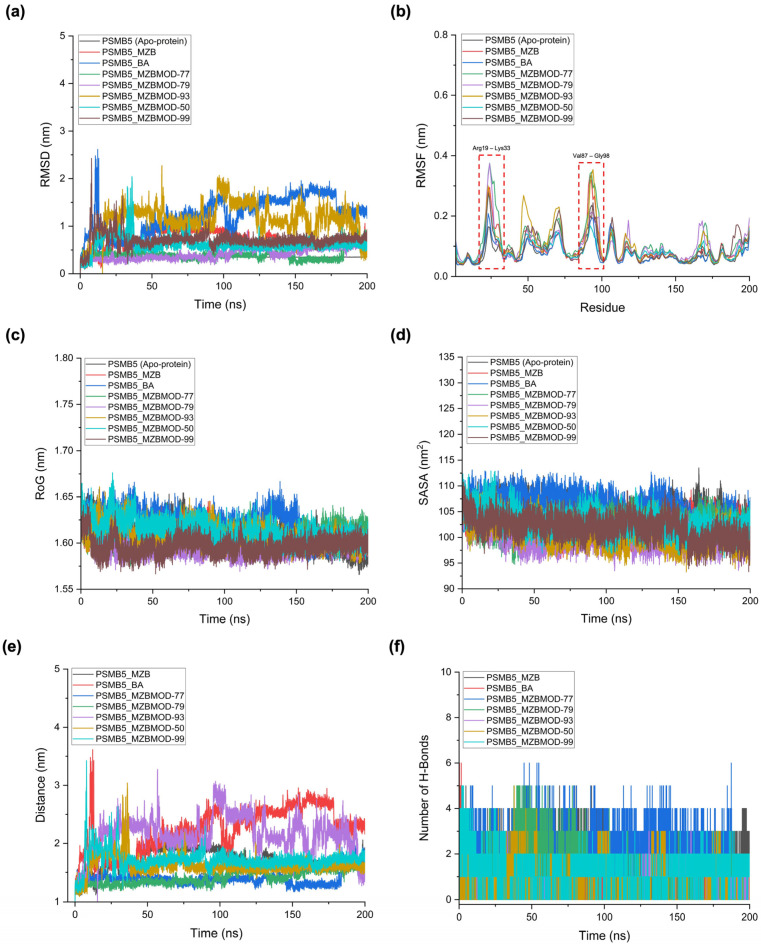
MD simulation results for PSMB5 in complex with Marizomib (MZB), Betulinic Acid (BA), and top-performing analogs over 200 ns of simulation. (**a**) Root mean square deviation (RMSD), reflecting the overall conformational stability of the protein–ligand complexes. (**b**) Root mean square fluctuation (RMSF) provides residue-level insights into backbone flexibility, particularly in the active-site regions. (**c**) Radius of gyration (RoG), indicating the degree of compactness and folding stability of the protein throughout the trajectory. (**d**) Solvent accessible surface area (SASA), showing changes in surface exposure and solvation upon ligand binding. (**e**) Ligand–protein center-of-mass distance, illustrating the persistence and dynamic retention of ligands within the binding cavity. (**f**) The number of hydrogen bonds represents the occupancy and stability of polar contacts sustaining protein–ligand interactions.

To assess the dynamic behavior and stability of PSMB5 in complex with MZB, BA, and the top-performing analogs, 200 ns MD simulations were performed. The analysis included RMSD, RMSF, radius of gyration (RoG), solvent-accessible surface area (SASA), ligand–protein center-of-mass distance, and hydrogen bond profiles (Table 5, Figure 6). The RMSD analysis (Figure 6a) demonstrated clear distinctions between stable and unstable complexes. Apo-PSMB5 exhibited minimal deviation (0.35 nm), reflecting inherent stability in the absence of ligands. The MZB complex displayed moderate deviations (0.70 nm), consistent with stable yet flexible interactions within the catalytic site. Among analogs, MZBMOD-77 and MZBMOD-79 showed the lowest RMSD values (0.40–0.42 nm), indicating exceptional structural stability and persistent binding throughout the trajectory. In contrast, MZBMOD-93 exhibited large fluctuations (1.15 nm), suggesting unstable engagement despite favorable docking energies. BA also showed relatively high RMSD (1.23 nm), which is consistent with its role as an activator, instead of tightly stabilizing the catalytic site as inhibitors do, BA induces conformational flexibility that may facilitate proteasome activation rather than inhibition.

The residue-level flexibility profiles (RMSF) (Figure 6b) further confirmed these trends. Most complexes maintained stable backbone fluctuations comparable to the apo-protein, with pronounced peaks consistently observed in the active-site loop regions Ala19–Lys33 and Val87–Gly98. These loops are critical for proteasome function, and their dynamic behavior reflects whether ligands induce inhibition or activation. Native MZB induced moderate fluctuations within these loops, consistent with its known inhibitory mechanism of disrupting local hydrogen-bonding stability to impair catalytic turnover. Similarly, MZBMOD-77 and MZBMOD-79 reproduced this effect, inducing slightly elevated flexibility in the loop regions, suggesting that they mimic the destabilizing behavior of MZB. This destabilization is supported by pharmacophore modeling, where these analogs established extensive hydrophobic and hydrogen-bonding contacts that interfere with loop rigidity. Other analogs, such as MZBMOD-50 and MZBMOD-99, also exhibited loop perturbations, though to a somewhat lesser extent, reinforcing their classification as inhibitor-like ligands. In contrast, BA (activator/agonist) reduced the amplitude of fluctuations in these same loop regions, promoting enhanced stabilization of the catalytic pocket. This is consistent with its mechanism of proteasome activation, where stabilization of the loop conformations facilitates substrate accommodation and turnover rather than inhibition. Interestingly, MZBMOD-93 produced broader but less consistent fluctuations, suggesting partial destabilization but without the same inhibitory efficiency observed for MZBMOD-77 and MZBMOD-79.

The radius of gyration (RoG) (Figure 6c) revealed overall compact folding across all complexes (1.59–1.62 nm), indicating that none of the ligands induced global structural collapse of PSMB5. Subtle distinctions, however, reflected their differing mechanisms. MZBMOD-79 maintained the lowest RoG (1.594 nm), suggesting a more compact and tightly folded proteasome structure when bound, consistent with its inhibitor-like conformational restriction of the active site and mirroring the reference engagement observed for native salinosporamide (VER). By contrast, BA induced a slight expansion (1.620 nm), a feature aligned with its agonist role of promoting loop stabilization and accommodating conformational flexibility necessary for proteasome activation. The SASA profiles (Figure 6d) echoed these findings. MZBMOD-79 and MZBMOD-93 reduced solvent exposure (99.8–100.8 nm^2^), indicating that inhibitor-like ligands restrict surface fluctuations and promote cavity closure, consistent with the canonical Thr1-centered binding network observed in the native VER reference. BA, however, increased the solvent-accessible surface area (105.9 nm^2^), consistent with a surface-exposed, dynamic environment that reflects loop stabilization rather than inhibitory occlusion.

The ligand–protein distance analysis (Figure 6e) further underscored these mechanistic distinctions. MZBMOD-77 (1.30 nm) and MZBMOD-79 (1.52 nm) remained persistently anchored within the catalytic cavity, effectively recapitulating the stable binding pattern of native VER and reinforcing their classification as inhibitors. Native MZB and other analogs such as MZBMOD-50 and MZBMOD-99 displayed intermediate stability (1.70–1.86 nm), consistent with their moderate inhibitory potential. In sharp contrast, MZBMOD-93 and BA drifted away from the cavity (≥2.0 nm). For BA, however, this cavity disengagement reflects its distinct activator function, in which it stabilizes active-site loop conformations from outside the canonical inhibitory pose rather than directly occluding the catalytic core. Finally, the hydrogen bond analysis (Figure 6f) highlighted the persistent engagement of key inhibitory analogs. MZBMOD-77 sustained the highest average number of hydrogen bonds (2.4), surpassing native MZB (1.9) and aligning with the interactions seen for native VER, indicating superior inhibitory potential. MZBMOD-79 formed fewer H-bonds (1.3), but this was compensated by extensive hydrophobic complementarity, consistent with its stable anchoring and favorable pharmacophore/FMO features. By contrast, MZBMOD-93, MZBMOD-50, and MZBMOD-99 formed fewer polar contacts (<1.0 on average), explaining their weaker dynamic stability. BA exhibited fewer direct H-bonds but appeared to reinforce hydrogen-bond stability within the loop regions (Ala19–Lys33 and Val87–Gly98), consistent with its agonist role of stabilizing loop conformations, in line with its distinct interaction mode relative to the inhibitory reference.

### 2.6. MM/PBSA Free Energy Analysis and Per-Residue Decomposition

**Figure 7:**
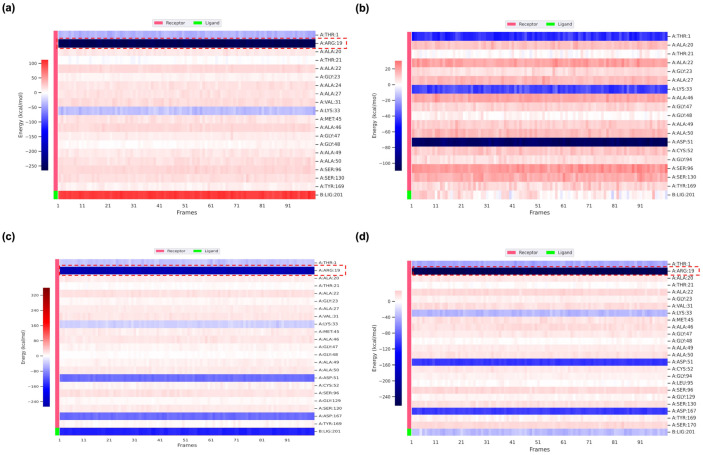
Heatmap of per-residue energy contributions (kcal/mol) in the ligand-binding domain of PSMB5. (**a**) PSMB5_MZB complex. (**b**) PSMB5_BA complex. (**c**) PSMB5_MZBMOD-77 complex. (**d**) PSMB5_MZBMOD-79 complex.

To further elucidate the thermodynamic determinants of ligand binding, MM/PBSA free energy calculations and per-residue decomposition analyses were performed for PSMB5 in complex with MZB, BA, and the top-performing analogs (Table 6, Figure 7). The binding free energies (ΔG_binding) revealed clear energetic distinctions between inhibitory MZB analogs and the agonist BA. Native MZB exhibited a modest binding free energy (−6.26 kcal/mol), while BA showed a comparable but slightly weaker profile (−5.60 kcal/mol), reflecting its non-inhibitory stabilizing interactions. Strikingly, MZBMOD-77 (−19.99 kcal/mol) and MZBMOD-79 (−18.79 kcal/mol) displayed markedly more favorable binding free energies, underscoring their enhanced inhibitory potential relative to the native scaffold. In contrast, MZBMOD-93 (−4.15 kcal/mol) exhibited the weakest binding, consistent with its unstable dynamics observed in MD simulations, while MZBMOD-50 (−11.00 kcal/mol) and MZBMOD-99 (−7.51 kcal/mol) occupied intermediate positions, suggesting moderate inhibitory capacity.

The per-residue energy decomposition maps (Figure 7a–d) provided residue-level insights into the molecular basis of these energetic trends. In the native MZB complex (Figure 7a), the most pronounced favorable contributions originated from the active-site loop residues Ala19–Lys33 and Val87–Gly98, which are known to regulate substrate entry and catalytic turnover. Within these regions, Arg19, Ala20, Lys33, and Ala50 stood out as the strongest contributors, underscoring their critical role in anchoring MZB within the β5 catalytic pocket. This pattern reflects the classical inhibitory mechanism of MZB, where binding disrupts local hydrogen-bond networks and loop flexibility, thereby impairing catalytic function. By contrast, BA (Figure 7b) presented a markedly different energetic landscape. The interaction profile was weaker and more diffuse, with limited stabilization from the key catalytic loop residues. Instead of focusing its binding energy at Arg19, Lys33, or Ala50, BA engaged more peripherally distributed residues, resulting in a diluted contribution pattern. This energetic footprint suggests that BA does not significantly destabilize loop hydrogen bonds. Rather, it appears to reinforce loop conformations, consistent with its agonist role, which involves maintaining structural integrity of the active-site loops and promoting proteasome activity rather than inhibition.

Among the MZB analogs, MZBMOD-77 (Figure 7c) exhibited the most striking inhibitory profile. The decomposition analysis revealed deep and localized binding energy wells centered on Arg19, Ala20, Lys33, and Ala49–Ala50, mirroring and amplifying the interaction hotspots observed in the MZB complex. This suggests that MZBMOD-77 not only mimics the native inhibitor but also establishes stronger and more persistent contacts that further destabilize loop hydrogen bonding. The enhanced engagement of Ala49–Ala50 is particularly notable, as this region is directly adjacent to the catalytic threonine residue, implying that MZBMOD-77 may exert stronger steric and electronic hindrance on proteasome function than the parent compound. Similarly, MZBMOD-79 (Figure 7d) displayed a robust but slightly more distributed interaction profile. Favorable contributions were again centered around Arg19, Ala20, and Lys33, with additional stabilization observed across broader loop residues. Unlike MZBMOD-77, which concentrated its energy contributions at specific catalytic hotspots, MZBMOD-79 engaged in a balanced interplay of polar and hydrophobic interactions, creating a more diffuse but still potent inhibitory footprint. This distribution likely explains its ability to maintain strong dynamic stability despite forming fewer direct hydrogen bonds, as observed in the MD simulations, where hydrophobic complementarity compensated for reduced polar contacts.

### 2.7. In Silico Pharmacokinetics and ADMET Profiling of Marizomib and Its Analogs

To complement the structural and energetic analyses, the pharmacokinetic and ADMET properties of MZB and its top-performing analogs were evaluated using in silico tools (Table 7). This analysis provided insights into drug-likeness, absorption, metabolism, and toxicity liabilities, thereby helping to prioritize analogs with the most favorable pharmacological profiles. Drug-likeness and physicochemical parameters showed that all analogs complied reasonably well with Lipinski’s rules, although their molecular weights were higher than that of native MZB (313.78 g/mol). MZBMOD-77 (481.58 g/mol) and MZBMOD-79 (407.50 g/mol) exhibited acceptable hydrogen bond donors (2) and acceptors (6), along with favorable lipophilicity (cLogP of 2.81 and 1.59, respectively), suggesting balanced hydrophobic–hydrophilic properties suitable for oral bioavailability. Compared to MZB (cLogP 0.51), these analogs demonstrated improved membrane permeability while maintaining optimal polar surface areas (PSA ≈ 100 Å^2^), which remained within the threshold compatible with good absorption and blood–brain barrier penetration.

Absorption and distribution parameters further supported the potential of MZBMOD-77 and MZBMOD-79. Both analogs exhibited superior predicted human oral absorption (100% and 90.7%, respectively), in contrast to native MZB (78.9%). Their high Caco-2 permeability values (1026.9 for MZBMOD-77 and 741.3 for MZBMOD-79) also indicated efficient intestinal absorption. Importantly, both analogs maintained favorable QPlogBB values (−0.397 and −0.497, respectively), suggesting moderate capacity for CNS penetration, which is relevant for proteasome inhibitors used in neurological malignancies. Distribution parameters such as plasma protein binding (QPlogKhsa) also highlighted improved interaction for MZBMOD-77 (0.105), indicating better systemic stability. Metabolism and safety predictions revealed a critical divergence between the analogs. While MZBMOD-77 and MZBMOD-79 were predicted to inhibit CYP3A4, raising the possibility of drug–drug interactions, their toxicity liabilities were markedly improved compared to native MZB. Unlike MZB, which showed mutagenic, tumorigenic, and reproductive toxicity risks, both MZBMOD-77 and MZBMOD-79 were predicted to be non-mutagenic, non-tumorigenic, and free of reproductive toxicity concerns. Irritancy was negligible for MZBMOD-79, and only low for MZBMOD-77, underscoring a significantly safer pharmacological profile. In contrast, MZBMOD-93 carried mutagenicity and irritancy risks, while MZBMOD-50 and MZBMOD-99 demonstrated lower oral absorption and less favorable distribution profiles.

Molecular flexibility and surface properties provided further support for the superior drug-like potential of MZBMOD-77 and MZBMOD-79. Both exhibited reduced molecular flexibility (0.323 and 0.308, respectively) compared to MZB (0.398), suggesting improved conformational stability during target engagement. Their favorable solvent accessible surface area (SASA) values and balance between hydrophobic (FOSA) and hydrophilic (FISA) components also implied better interactions with both membrane environments and aqueous systems. Taken together, these pharmacokinetic and ADMET evaluations reinforce MZBMOD-77 and MZBMOD-79 as the most promising MZB analogs, combining potent inhibitory mechanisms (as shown in the structural and energetic analyses) with optimized bioavailability and markedly improved safety profiles compared to native MZB. While CYP3A4 inhibition warrants further investigation, their overall balance of efficacy, absorption, and reduced toxicity strongly positions them as lead candidates for preclinical development as next-generation proteasome inhibitors.

## 3. Discussion

This work assembled a complete in silico (design–evaluate–prioritize) pipeline around the β5 (chymotrypsin-like) catalytic subunit of the 20S proteasome to explore why MZB (salinosporamide A) shows attractive CNS pharmacology yet mixed clinical efficacy in GBM, and whether rational analogs could improve the profile. Our study advances the field by implementing an integrated computational pipeline that combines rational 3D structural design, MM2 energy minimization, docking, pharmacophore modeling, long-timescale (200 ns) MD simulations, MM/PBSA free energy calculations, and multi-tool ADMET evaluation, enabling simultaneous assessment of binding stability, selectivity, pharmacophoric complementarity, electronic properties, and drug-likeness—an approach not comprehensively applied in prior MZB analog studies. Importantly, we explicitly considered translationally relevant features, including β5 selectivity versus off-target β1/β2 subunits and pharmacokinetic liabilities, while mechanistic insights from loop dynamics and per-residue energy decomposition reveal key interactions mediating inhibitory potency. Our results converge on two fused/polycyclic chemotypes (MZBMOD-77 and MZBMOD-79) that consistently outperform native MZB across docking, pharmacophore agreement, long-timescale MD, MM/PBSA free energies, and several ADMET surrogates. These computational findings dovetail with the experimentally established mechanism of MZB, an irreversible, β-lactone warhead targeting the N-terminal Thr1 of β subunits, and with the observation that BBB penetration distinguishes MZB from earlier proteasome inhibitors [23,24]. Docking plus 3D pharmacophores recapitulated the canonical Arg19/Thr21/Gly23/Gly47 hydrogen-bond network stabilizing MZB in the β5 pocket and correctly oriented the β-lactone–γ-lactam core. MZBMOD-77 and MZBMOD-79 preserved this network while extending into a hydrophobic cleft, increasing van der Waals complementarity without sacrificing the directional H-bonding seen in MZB. In contrast, BA, included as a 20S activator/agonist control, bound more weakly and with fewer persistent polar contacts, as expected for small-molecule 20S activators that favor pore-opening/stabilizing conformations rather than catalytic—site disruption. 

The MD results sharpen this mechanistic distinction. Inhibitory complexes (MZB, MZBMOD-77, MZBMOD-79) showed low RMSDs, short ligand–protein distances, and, crucially, elevated RMSF in two active-site loop segments (Ala19–Lys33 and Val87–Gly98), indicating disruption of the local H-bond lattice that normally stabilizes the β5 pocket. BA did the opposite; it dampened fluctuations in the same loops and displayed larger ligand drift, an activator-like stabilization inconsistent with covalent active-site engagement. These patterns were mirrored in H-bond occupancies (higher and more persistent for MZBMOD-77; fewer but compensated by hydrophobics for MZBMOD-79), and in SASA/RoG trends (MZBMOD-79 slightly compacts the core; BA modestly expands it). Together, the dynamics provide a functional signature, loop destabilization for inhibitors versus loop stabilization for activators, that accords with structural/mechanistic studies showing MZB’s irreversible blockade of catalytic threonines and contrasts with small-molecule 20S activators that bias the gate/open states [16,25]. MM/PBSA ranked the analogs in line with dynamics: MZBMOD-77 (ΔG_bind ≈ −20 kcal/mol) and MZBMOD-79 (≈ −19 kcal/mol) were markedly more favorable than MZB (≈ −6 kcal/mol). Per-residue decomposition highlighted Arg19, Ala20, Lys33, and Ala49–Ala50 as dominant contributors, a set that matches the chemistry of lactone/lactam anchoring near Thr1 and the adjacent β-strands lining the pocket. By contrast, BA exhibited diffuse, weaker contributions from the identical loop residues, again consistent with an agonist paradigm rather than covalent-like inhibitory engagement. These residue-level hot spots agree with high-resolution structural studies of salinosporamide–proteasome complexes, which show covalent Thr1 acylation and extensive contacts with nearby β5 residues [16,23]. Importantly, we acknowledge a key limitation of the present computational strategy. MZB and related β-lactone proteasome inhibitors act through covalent modification of the catalytic Thr1γ. The current docking, pharmacophore, MD, and MM/PBSA analyses capture only the pre-covalent non-covalent complex, which represents the recognition step but not the full inhibitory mechanism. While this non-covalent view is valuable for prioritizing analogs, it remains a partial description of the true pharmacology. More rigorous treatments, such as QM/MM covalent docking or free-energy perturbation (FEP+) calculations, would be required to fully model the covalent bond formation and energy landscape of Thr1 acylation. These resource-intensive methods were beyond the present study’s scope, but they constitute a critical next step to validate and extend our findings.

Encouragingly, both MZBMOD-77 and MZBMOD-79 retained CNS-compatible polarity (QPlogBB ≈ −0.40 to −0.50; similar to MZB) and high predicted oral absorption (≈90–100%), while removing several in silico toxicity alerts flagged for MZB (mutagenicity/tumorigenicity/reproductive effects). Potential liabilities emerged as well: CYP3A4 inhibition was predicted for both, which could drive drug–drug interactions and would need to be engineered out or clinically managed. Overall, the ADMET profile suggests that MZBMOD-77 and MZBMOD-79 could preserve MZB’s BBB penetration, a property emphasized in recent structural/biophysical work, while improving safety margins, a key requirement given the negative outcome of the large phase III GBM trial that combined MZB with standard chemoradiation. Our observations are congruent with classic salinosporamide SAR: modifications that rigidify the bicyclic core and optimize occupancy of hydrophobic sub-pockets generally enhance β5 potency, provided they preserve the electrophilic geometry for Thr1 acylation. Early crystallography and SAR around salinosporamide A showed that β-lactone opening and ensuing cyclic rearrangement lock the inhibitor in place and that appropriately substituted rings can increase binding while controlling reactivity [15,16]. The best current analogs (MZBMOD-77 and MZBMOD-79) appear to exploit the same design logic computationally, fused systems that rigidify the scaffold, preserve the H-bonding motif, and increase desolvation/hydrophobic gains. 

## 4. Materials and Methods

### 4.1. Three-Dimensional Structure Modifications and MM2 Energy Minimization

The three-dimensional structures of MZB and its designed derivatives were constructed using Chem3D Ultra 22.0 (PerkinElmer, Waltham, MA, USA). Structural modifications were rationally designed with the dual aim of enhancing the binding interactions of MZB with the human proteasome subunit beta type-5 (PSMB5) and reducing potential off-target liabilities. To ensure structural feasibility and conformational stability, each derivative underwent MM2 energy minimization, a molecular mechanics method that optimizes bond lengths, bond angles, torsional strain, and steric hindrance, thereby refining the geometry into a low-energy, physically realistic conformation [26,27]. The modification strategies were systematically categorized into six major groups based on their chemical rationale and anticipated impact on pharmacological properties: (i) halogenation, involving the substitution or addition of halogen atoms (Cl, Br, F, I) to modulate lipophilicity and binding affinity [28,29]; (ii) alkyl/aryl substitutions, including chain extension or introduction of phenyl and aryl groups to improve hydrophobic interactions and π-stacking [30,31]; (iii) hydroxyl/ether modifications, such as hydroxyl relocation, methoxy substitution, or ether linkages, to adjust hydrogen-bonding capacity and solubility [32,33]; (iv) lactone/lactam ring modifications, targeting the γ-lactam–β-lactone bicyclic core through ring expansion, contraction, or reconfiguration to fine-tune covalent warhead reactivity [14,34]; (v) ester modifications, involving the incorporation or variation in ester functionalities (acetates, methyl esters, carbamates) to alter metabolic stability and polarity [35,36]; and (vi) fused/polycyclic/complex cyclized derivatives, where additional fused or bridged ring systems were introduced to rigidify the scaffold and explore novel binding orientations [37,38]. These modification strategies provided a diverse library of candidate structures with the potential to optimize proteasome inhibition while mitigating the toxicity and limited clinical efficacy observed with native MZB.

### 4.2. Three-Dimensional Structure Alignment and Similarity Analysis

To systematically evaluate structural consistency and conformational diversity among MZB and its designed analogs, a two-step similarity assessment was performed combining both 3D structural alignment and molecular fingerprint-based comparisons. First, conformational alignment was conducted using the superimpose tool in BIOVIA Discovery Studio 2024 (Dassault Systèmes, Vélizy-Villacoublay, France) [39], which enabled visualization of atomic overlap and pharmacophoric feature distribution across the analog series. This allowed direct inspection of backbone preservation and functional group variation relative to native MZB. To complement this structural assessment with a quantitative approach, pairwise molecular similarities were computed using the Tanimoto coefficient, derived from cheminformatics fingerprints. Two fingerprinting strategies were applied: (i) FP2 (path-based), which captures linear atom paths up to a predefined length [40,41], and (ii) Morgan fingerprints (ECFP4), which encode circular atom environments, offering complementary sensitivity to local structural modifications [42,43]. The similarity calculations were implemented in Python (https://github.com/donidermawan/pharmaceutics_MZB_analogs (accessed on 1 September 2025)) using the RDKit toolkit, with custom scripts developed for both bar chart ranking of analog similarities against native MZB and hierarchical clustering heatmaps for full similarity matrices. These analyses established a comprehensive framework to quantify structural conservation, identify divergent modifications, and prioritize analogs for subsequent docking and dynamics evaluations. 

### 4.3. Molecular Docking Simulations and Binding Affinity Analysis

Molecular docking was performed to characterize the interactions between MZB, its structural analogs, and the PSMB5. The primary goal was to identify key amino acid residues mediating ligand–protein binding, define the types of intermolecular forces contributing to affinity, and compare binding orientations across the designed derivatives to evaluate how specific modifications influence binding performance. The crystal structure of PSMB5 (PDB ID: 8BZL [22], chain L; resolution 2.14 Å) was retrieved from the RCSB Protein Data Bank. Prior to docking, the receptor structure was refined using Swiss-PdbViewer v4.1 (Swiss Institute of Bioinformatics, Lausanne, Switzerland) [44], which involved removing crystallographic water molecules, performing energy minimization, and repairing missing side chains to yield an optimized structure suitable for ligand docking. The active site and binding pockets were annotated using PDBSum (European Bioinformatics Institute, Cambridge, UK) [45], ensuring accurate localization of functionally relevant residues. To provide a comparative reference, Betulinic Acid (BA), a known activator of human 20S proteasomes [46], was included in the study, and its geometry was optimized via MM2 energy minimization under the same conditions as MZB and its analogs.

Docking simulations were carried out using the HADDOCK v2.4 platform (University of Utrecht, Utrecht, The Netherlands), which differs from conventional docking tools by integrating ambiguous interaction restraints (AIRs) derived from experimental or computational data, thereby enhancing prediction accuracy and biological relevance [47,48]. The standalone interface enabled advanced customization of docking parameters, including both geometric and energetic constraints, to improve reliability. Docking outputs were evaluated through cluster analysis and HADDOCK scoring functions. Binding models were grouped into clusters, with the largest cluster considered the most representative due to its reproducibility and stability. The HADDOCK score, a composite of van der Waals, electrostatics, desolvation energy, and buried surface area contributions, was used to rank docking poses. The top-ranked complex with the most favorable score was selected as the representative binding model. To complement docking outcomes, binding free energies (ΔG, kcal/mol) were further estimated using the PRODIGY server, which calculates ΔG based on structural determinants such as intermolecular contacts, buried surface area, and desolvation effects [49]. This combined strategy provided both qualitative insight into binding modes and quantitative predictions of ligand–protein affinity, enabling robust comparison of MZB analogs and identification of promising candidates for further study. In addition to β5, we also performed supplementary docking against the β1 (PSMB6; PDB ID: 5LE5, chain N [50]) and β2 (PSMB7; PDB ID: 5LE5, chain H [50]) subunits. These analyses were not the central focus of the study but were conducted to address translational concerns regarding off-target engagement and systemic toxicity. Comparative ∆∆G values were calculated to evaluate whether the designed analogs exhibited preferential affinity for β5 relative to β1 and β2.

### 4.4. HOMO–LUMO Analysis of MZB Analogs

The frontier molecular orbital (FMO) characteristics of MZB and its designed analogs were investigated using density functional theory (DFT) implemented through a custom Python workflow. All molecular structures were fully optimized at the PBE0/6-31G(d) level of theory, which offers a balance between computational efficiency and reliable electronic structure prediction [51]. Convergence criteria were set to tight self-consistent field (SCF) thresholds, with an energy tolerance of 10^−6^ Ha and a maximum gradient of 10^−3^ Ha/Bohr, ensuring accurate and stable optimization [52]. Following geometry optimization, single-point energy calculations were conducted at the same level to obtain orbital eigenvalues. The script automatically identified the highest occupied molecular orbital (HOMO) and lowest unoccupied molecular orbital (LUMO) and computed the energy gap (ΔE = E_LUMO −E_HOMO). This parameter is a critical descriptor of electronic excitation potential, molecular stability, and chemical reactivity [53]. To visualize orbital delocalization, frontier orbital iso-surfaces were generated using standard visualization tools, with an isovalue of 0.02 a.u. [54,55], enabling assessment of how specific structural modifications affect electron density distribution across the molecular framework. This computational protocol provided a consistent and reproducible framework for comparing the electronic properties of MZB analogs, thereby supporting rationalization of their reactivity and potential activity as selective PSMB5 inhibitors.

### 4.5. Three-Dimensional Pharmacophore Modeling

Three-dimensional (3D) pharmacophore modeling was performed to elucidate the key molecular features underlying the interactions of MZB and its analogs with PSMB5. This strategy provides a structural basis for understanding how distinct chemical functionalities contribute to receptor recognition and ligand affinity. Pharmacophore models were generated using LigandScout version 4.5 (Inte:Ligand, Vienna, Austria) [56], which identifies and maps critical interaction features such as hydrogen bond donors (HBDs), hydrogen bond acceptors (HBAs), hydrophobic moieties, and electrostatic interaction centers. These elements represent the fundamental determinants of ligand–protein binding specificity and stability. By employing LigandScout’s automated feature detection and alignment algorithms, comparative pharmacophore models were constructed for MZB and its analogs. This analysis facilitated the identification of conserved interaction motifs and highlighted structural modifications that either preserved or enhanced key pharmacophoric features, providing insights into the molecular basis of binding optimization.

### 4.6. Molecular Dynamics (MD) Simulation for Structural Stability and Interaction Analysis

The MD simulations were conducted to explore the conformational dynamics, structural stability, and interaction patterns of MZB and its analogs in complex with PSMB5. All simulations were performed using GROMACS version 2025.2 [57]. Ligand topologies were generated with the General Amber Force Field (GAFF2), and atomic partial charges were assigned via the AM1-BCC method [58,59] using acpype integrated with AmberTools21. The receptor was parameterized with the AMBER99SB force field, ensuring consistency in treating bonded and non-bonded interactions. The protein–ligand complexes were solvated in a dodecahedral box with the SPC water model, maintaining a minimum distance of 1.4 nm between solute atoms and box boundaries. Periodic boundary conditions (PBC) were applied in all directions, and the systems were neutralized with counterions, followed by the addition of 100 mM NaCl to mimic physiological ionic strength. Non-bonded interactions were handled with a 1.2 nm cutoff for van der Waals forces, while long-range electrostatics were calculated with the Particle Mesh Ewald (PME) method [60]. Energy minimization was performed using the steepest descent algorithm until the maximum force was below 1000 kJ/mol/nm, removing steric clashes and optimizing the starting structure. Equilibration was carried out in two steps: (i) NVT ensemble for 100 ps, applying positional restraints to stabilize temperature at 310 K using the Berendsen thermostat, followed by (ii) NPT ensemble for 100 ps, equilibrating pressure at 1 bar with the Berendsen barostat [59,61]. Subsequently, unrestrained 200 ns production runs were performed, with temperature maintained using the V-rescale thermostat and pressure regulated via the Parrinello–Rahman barostat. Trajectories were recorded every 2 fs for post-simulation analyses. Trajectory processing and structural evaluation focused on monitoring root mean square deviation (RMSD), root mean square fluctuation (RMSF), radius of gyration (RoG), solvent-accessible surface area (SASA), hydrogen bonding patterns, and binding pocket dynamics. Interaction profiles were analyzed using PyMOL version 3.1.3 (Schrödinger LLC, New York, USA) [62], BIOVIA Discovery Studio 2024 (Dassault Systèmes, San Diego, USA) [39], and UCSF ChimeraX (University of California, San Francisco, USA) [63], enabling both quantitative measurements and qualitative visualization of the ligand–protein dynamics.

### 4.7. Molecular Mechanics/Poisson–Boltzmann Surface Area (MM/PBSA) Calculations

The binding free energies of MZB and its analogs in complex with PSMB5 were estimated using the MM/PBSA method, applied to equilibrated frames extracted from the MD trajectories. This approach integrates gas-phase molecular mechanics energies, solvation effects, and entropic contributions to provide a comprehensive evaluation of ligand–protein binding affinity [64]. For each snapshot, three principal energetic terms were calculated: (i) molecular mechanics energy in the gas phase (electrostatic and van der Waals interactions), (ii) solvation free energy, composed of a polar component derived from the Poisson–Boltzmann continuum model and a nonpolar component estimated from solvent-accessible surface area (SASA), and (iii) entropic contributions accounting for the conformational flexibility of both ligand and receptor. The Single-Trajectory Protocol (STP) was adopted, whereby the receptor, ligand, and complex free energies were computed from the same MD trajectory. This strategy reduces computational cost and improves consistency by assuming limited conformational rearrangements upon binding [65,66,67]. Free energy calculations were carried out using gmx_MMPBSA version 1.6.4, integrated with the GROMACS simulation framework [68]. The overall binding free energy (ΔG_binding) was determined according to the standard thermodynamic cycle:

ΔG_binding = ΔG_complex − ΔG_ligand − ΔG_receptor

where ΔG_complex is the total free energy of the solvated protein–ligand complex, ΔG_ligand represents the free energy of the unbound ligand in solution, and ΔG_receptor denotes the free energy of the isolated receptor. The calculated ΔG_binding values provided a quantitative basis for comparing analogs and identifying modifications that confer improved binding affinity and interaction stability relative to native MZB.

### 4.8. In Silico Pharmacokinetics and ADMET Evaluation

The pharmacokinetic profiles and drug-likeness of MZB analogs were evaluated using a multi-platform computational strategy. SwissADME (Swiss Institute of Bioinformatics, Lausanne, Switzerland) [69] was first applied to calculate key physicochemical descriptors, including molecular weight, lipophilicity (MlogP), HBD, HBA, and topological polar surface area (TPSA). These parameters were compared against Lipinski’s Rule of Five, a benchmark for estimating oral bioavailability. Analogs meeting these criteria were considered more likely to demonstrate favorable absorption and permeability, supporting their potential as orally active drugs. In addition, SwissADME predicted interactions with cytochrome P450 (CYP) isoenzymes, offering insights into metabolic stability, clearance, and possible drug–drug interactions. To complement these assessments, DataWarrior version 6.4.1 (OpenMolecules, Karlsruhe, Germany) [70] was used to evaluate drug-likeness and toxicity risk. This tool integrates molecular descriptors with predictive models to identify potential toxicological concerns, including mutagenicity, tumorigenicity, reproductive toxicity, and irritant properties, thereby flagging structural liabilities at an early stage. A more detailed pharmacokinetic and ADMET profiling was performed using QikProp (Schrödinger, New York, NY, USA) [71]. QikProp computed advanced descriptors related to absorption, distribution, metabolism, and excretion (ADME), including solvent-accessible surface area (SASA), hydrophobic SASA (FOSA), hydrophilic SASA (FISA), percentage of predicted human oral absorption, QPlogHERG (hERG channel inhibition, a measure of cardiotoxicity), QPPCaco (Caco-2 cell permeability), QPlogBB (blood–brain barrier penetration), QPPMDCK (MDCK cell permeability), QPlogKp (skin permeability), and QPlogKhsa (serum protein binding). Collectively, these evaluations provided a robust computational framework for prioritizing analogs with optimized pharmacokinetic behavior, reduced toxicity risks, and improved drug-likeness relative to MZB.

## 5. Limitations and Future Works

Despite the promising findings, several limitations of this study should be recognized. First, while molecular docking, pharmacophore modeling, MD simulations, and MM/PBSA free energy calculations provided important insights into the binding stability and energetic landscape of Marizomib and its analogs, these methods primarily capture the noncovalent pre-inhibition interactions. In reality, Marizomib acts through a covalent modification of the β5 catalytic threonine, and this irreversible step was not explicitly modeled here. As such, our current results should be interpreted as indicators of relative binding affinity and stability rather than definitive measures of inhibitory potency. Future work incorporating QM/MM simulations or alchemical FEP could more accurately account for covalent bond formation and predict reactivity. Second, the initial docking experiments relied on a single crystal structure of PSMB5 (PDB ID: 8BZL) as the receptor conformation. While this approach established a consistent baseline for analog comparison, it does not fully capture the conformational plasticity of active site loops. Although 200 ns MD simulations were subsequently performed to address loop flexibility and ligand stability, we acknowledge that an ensemble docking approach, using cluster-representative conformations extracted from MD trajectories, would provide a more physiologically relevant representation of proteasome–ligand interactions. Future work incorporating ensemble docking is therefore warranted to complement our current single-structure docking strategy.

Third, the ADMET predictions, while encouraging, remain in silico approximations. For instance, the predicted CYP3A4 inhibition by MZBMOD-77 and MZBMOD-79 raises a potential liability for drug–drug interactions (DDIs), as co-administered CYP3A4 substrates could experience altered plasma exposure. QPlogHERG predictions highlight the need for experimental validation of potential cardiotoxic liabilities. Moreover, off-target toxicity, metabolic stability, and bioavailability cannot be fully captured through computational models alone. To experimentally test our predictions, in vitro enzymatic assays measuring β5 catalytic activity will be performed to confirm inhibitory potency, cytotoxicity assays in relevant GBM cell lines will assess cellular efficacy and safety, and in vivo or ex vivo pharmacokinetic studies in animal models will validate BBB penetration, metabolism, and clearance. Additionally, while BA was included as a control activator, future studies could incorporate clinically approved proteasome inhibitors such as carfilzomib and bortezomib as comparative references to contextualize analog performance. Furthermore, the chemical synthesis feasibility of MZBMOD-77 and MZBMOD-79 will be evaluated, including assessments of synthetic stability, stereochemical complexity, and scalability, to ensure they can advance to preclinical testing.

Finally, although MZBMOD-77 and MZBMOD-79 demonstrated favorable physicochemical properties and predicted BBB permeability, these findings require confirmation in preclinical pharmacokinetic studies. Given that BBB penetration is central to the clinical utility of Marizomib, ensuring that these analogs retain efficient CNS delivery will be a critical next step before considering translational applications. Future efforts should therefore priori-tize (i) chemical synthesis of the most promising analogs, particularly MZBMOD-77 and MZBMOD-79, followed by in vitro biochemical assays to validate β5 inhibitory activity and cytotoxicity, (ii) ADMET validation using in vitro and in vivo systems to confirm pharmacokinetic behavior, BBB penetration, and safety profiles, and (iii) extended computational modeling, including ensemble docking, covalent inhibition simulations, and longer MD trajectories, to strengthen the mechanistic understanding. These steps will provide a robust foundation for advancing MZBMOD-77 and MZBMOD-79 as next-generation proteasome inhibitors with improved therapeutic potential for CNS malignancies.

## 6. Conclusions

In conclusion, this study employed an integrative in silico framework that combined molecular docking, pharmacophore modeling, molecular dynamics simulations, MM/PBSA free energy calculations, and ADMET predictions to systematically evaluate MZB and its structural analogs as modulators of the proteasome β5 subunit. Our findings demonstrate that while native MZB binds stably and disrupts active-site loop dynamics, two designed analogs, MZBMOD-77 and MZBMOD-79, exhibited superior binding free energies, enhanced hydrogen-bonding persistence, and favorable pharmacophore complementarity, effectively mimicking and amplifying the inhibitory mechanism of MZB. Dynamic simulations further highlighted the capacity of these analogs to destabilize catalytic loop regions (Ala19–Lys33 and Val87–Gly98), underscoring their potential as next-generation proteasome inhibitors. Per-residue energy decomposition revealed key hotspot residues, including Arg19, Ala20, Lys33, and Ala50, mediating strong interactions with MZBMOD-77 and MZBMOD-79, providing structural insights into their enhanced potency. Importantly, ADMET profiling suggested that these analogs retained drug-like properties and showed improved safety and pharmacokinetic profiles compared to native MZB, with high predicted oral absorption and acceptable BBB permeability. These results identify MZBMOD-77 and MZBMOD-79 as the most promising candidates for further development. While additional experimental validation is warranted, the present study establishes a strong computational foundation to guide the rational optimization, synthesis, and preclinical evaluation of novel proteasome inhibitors with potential therapeutic applications in oncology.

## Figures and Tables

**Table 1:** Representative structural modifications of Marizomib (MZB) analogs showing Tanimoto similarity scores, SMILES notation, and 2D representations. The analogs listed here were selected based on interaction similarity to native MZB. The complete set of 100 designed analogs is provided in Supplementary Data S1.

Molecule	Modification Category	Tanimoto Similarity (FP2)	SMILES	2D Structure
MZB	N/A	1.000	C[C@]12[C@H](C(=O)N[C@]1(C(=O)O2)[C@H]([C@H]3CCCC=C3)O)CCCl	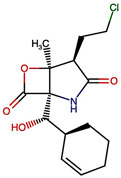
MZBMOD-1	Halogenation	0.927	C[C@@]12OC(=O)[C@@]1(NC(=O)[C@@H]2CCBr)[C@@H](O)[C@H]1CCCC=C1	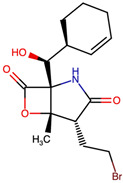
MZBMOD-4	Halogenation	0.909	C[C@@]12OC(=O)[C@@]1(NC(=O)[C@@H]2CCCCl)[C@@H](O)C1CCCCC1	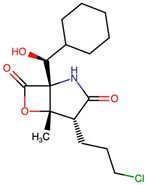
MZBMOD-22	Alkyl/Aryl Substitutions	0.601	CC(C)COC(=O)C1C2OC3(CN(C4CCCC4)C(=O)C13)C=C2	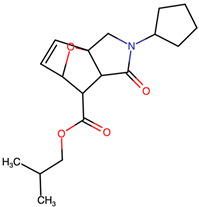
MZBMOD-25	Alkyl/Aryl Substitutions	0.847	CC1=CC=C(C=C1)S(=O)(=O)OCC[C@H]1C(=O)N[C@@]2([C@@H](O)[C@H]3CCCC=C3)C(=O)O[C@@]12C	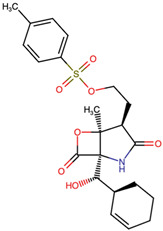
MZBMOD-36	Hydroxyl/Ether Modifications	0.900	C[C@@]12OC(=O)[C@@]1(NC(=O)[C@@H]2CCCl)[C@@H](O)[C@H]1CCC[C@H]2O[C@@H]12	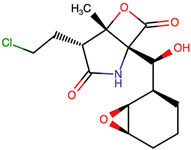
MZBMOD-38	Hydroxyl/Ether Modifications	0.721	COC(=O)CCSC(=O)[C@@]1(NC(=O)[C@@H]2CCO[C@]12C)[C@@H](O)[C@H]1CCCC=C1	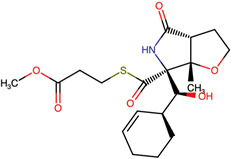
MZBMOD-50	Lactone/Lactam Ring Modifications	0.626	C[C@H]1C=C[C@H]2CCCC[C@@H]2[C@H]1C(=O)[C@H]1[C@@H]2OC(=O)[C@@H]3CCN(C1=O)[C@]23O	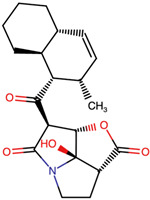
MZBMOD-56	Lactone/Lactam Ring Modifications	0.565	CCOC(=O)CN1CC23OC(C=C2)C(C3C1=O)C(=O)NC1CCCC1	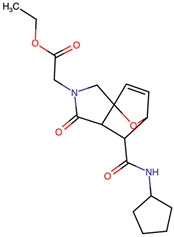
MZBMOD-68	Ester Modifications	0.449	CCOC(=O)[C@H]1CCCN1C(=O)C(=O)C1(CCCCC1)OC	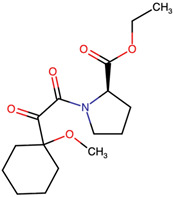
MZBMOD-72	Ester Modifications	0.446	CCOC(=O)C1CCCCN1C(=O)C(=O)C1(CCCCC1)OC	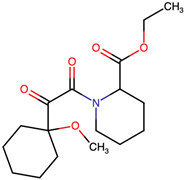
MZBMOD-77	Fused/Polycyclic/Complex Cyclized	0.626	C[C@H]1[C@H]2[C@H](CC3=CC=CC=C3)NC(=O)[C@]22OC(=O)CCC(C)C(=O)C(C)(O)C/C=C/[C@H]2[C@@H]2O[C@]12C	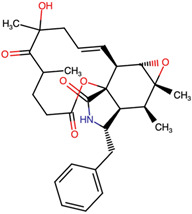
MZBMOD-79	Fused/Polycyclic/Complex Cyclized	0.542	CC(C)[C@H](NC(C)=O)C(=O)O[C@H]1CCC(C)(C)[C@@H]2CC=C3C(=O)OC[C@]3(O)[C@@]12C	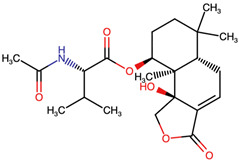

**Table 2:** Comparative docking results of Marizomib (MZB), Betulinic Acid (activator), and top-performing analogs with PSMB5, including binding affinities and interaction energy components. The complete molecular docking results are presented in Supplementary Data S2.

Complex	HADDOCK Score (a.u.)	Binding Energy (kcal/mol)	Van der Waals Energy	Electrostatic Energy	Desolvation Energy	RMSD	Hydrogen Bonds
PSMB5_MZB	−27.1 ± 0.4	−7.13	−22.3 ± 0.4	−10.1 ± 1.4	−3.8 ± 0.2	0.2 ± 0.0	Arg19, Thr21, Gly23, Gly47
PSMB5_BA	−22.4 ± 0.9	−6.98	−16.6 ± 0.8	−9.1 ± 5.3	−2.5 ± 0.4	0.2 ± 0.0	Gly47
PSMB5_MZBMOD-77	−34.8 ± 0.4	−8.09	−29.4 ± 0.5	−14.0 ± 2.6	−4.0 ± 0.5	0.1 ± 0.1	Arg19, Thr21, Gly23, Gly47
PSMB5_MZBMOD-79	−33.8 ± 0.4	−7.83	−27.0 ± 0.5	−37.5 ± 1.8	−3.1 ± 0.1	0.1 ± 0.1	Arg19, Thr21, Gly23, Gly47
PSMB5_MZBMOD-93	−28.2 ± 0.2	−7.39	−22.1 ± 0.6	−42.2 ± 3.3	−2.1 ± 0.1	1.5 ± 0.0	Arg19, Thr21, Gly23, Gly47
PSMB5_MZBMOD-50	−33.3 ± 0.2	−7.37	−29.0 ± 0.3	−20.6 ± 5.1	−2.3 ± 0.1	0.1 ± 0.1	Thr1, Arg19, Thr21, Gly47
PSMB5_MZBMOD-99	−28.7 ± 1.4	−7.30	−27.0 ± 1.0	−1.3 ± 7.2	−1.6 ± 0.3	0.2 ± 0.0	Arg19, Thr21, Gly47

**Table 3:** Molecular interaction profiles of PSMB5 with Marizomib (MZB), Betulinic Acid (BA), and top-performing analogs. The complete list of molecular interactions can be seen in Supplementary Data S3.

Complex	CC	CO	CN	CX	OO	OX	NO	NN	NX	XX
PSMB5_MZB	1363	852	521	21	128	6	132	24	2	0
PSMB5_BA	1269	793	511	33	91	4	70	0	0	0
PSMB5_MZBMOD-77	1950	1269	687	34	184	9	179	21	1	0
PSMB5_MZBMOD-79	1620	1104	588	28	174	11	170	17	1	0
PSMB5_MZBMOD-93	1324	838	535	23	131	10	142	32	1	0
PSMB5_MZBMOD-50	1426	1040	533	18	189	9	184	25	2	0
PSMB5_MZBMOD-99	1573	1101	608	30	169	5	181	35	2	0

**Table 4:** Frontier molecular orbital energies, HOMO–LUMO gap, and dipole moments of Marizomib (MZB) and its best-performing analogs.

Molecule	HOMO (eV)	LUMO (eV)	Gap (eV)	Dipole (D)
MZB	−6.76	−0.81	5.95	6.43
MZBMOD-77	−5.95	−0.35	5.60	2.72
MZBMOD-79	−6.80	−1.57	5.23	8.26
MZBMOD-93	−6.17	−1.24	4.92	2.91
MZBMOD-50	−6.52	−0.95	5.57	1.58
MZBMOD-99	−6.41	−1.08	5.33	7.88

**Table 5:** MD simulation parameters of PSMB5 complexes with Marizomib (MZB), Betulinic Acid (BA), and top-performing analogs over 200 ns of simulations, including RMSD, RMSF, RoG, SASA, ligand–protein center-of-mass distance, and number of hydrogen bond interactions.

Complex	Average RMSD (nm)	Average RMSF (nm)	Average RoG (nm)	Average SASA (nm^2^)	Average Distance (nm)	Number of Hydrogen Bonds Between the Ligand-Receptor
PSMB5 (Apo-protein)	0.347 ± 0.015	0.078 ± 0.038	1.608 ± 0.014	104.064 ± 2.283	N/A	N/A
PSMB5_MZB	0.702 ± 0.174	0.093 ± 0.053	1.606 ± 0.009	102.644 ± 2.027	1.864 ± 0.429	1.863 ± 0.781
PSMB5_BA	1.226 ± 0.370	0.076 ± 0.034	1.620 ± 0.014	105.868 ± 2.528	2.317 ± 0.865	0.414 ± 0.751
PSMB5_MZBMOD-77	0.396 ± 0.114	0.111 ± 0.064	1.606 ± 0.008	102.489 ± 1.770	1.303 ± 0.398	2.416 ± 0.864
PSMB5_MZBMOD-79	0.419 ± 0.122	0.099 ± 0.051	1.594 ± 0.008	99.847 ± 1.980	1.523 ± 0.547	1.300 ± 0.720
PSMB5_MZBMOD-93	1.146 ± 0.317	0.100 ± 0.061	1.609 ± 0.011	100.798 ± 2.267	2.018 ± 0.214	0.642 ± 0.536
PSMB5_MZBMOD-50	0.624 ± 0.155	0.086 ± 0.036	1.613 ± 0.012	103.070 ± 2.320	1.741 ± 0.152	0.708 ± 1.025
PSMB5_MZBMOD-99	0.711 ± 0.153	0.093 ± 0.047	1.596 ± 0.009	101.919 ± 2.242	1.821 ± 0.427	0.617 ± 0.807

**Table 6:** MM/PBSA binding free energies (ΔG_binding) of Marizomib (MZB), Betulinic Acid (BA), and top-performing analogs in complex with PSMB5.

Complex	MM/PBSA Free Binding Energy ΔG_Binding (kcal/mol)
PSMB5_MZB	−6.26 ± 4.08
PSMB5_BA	−5.60 ± 6.01
PSMB5_MZBMOD-77	−19.99 ± 4.75
PSMB5_MZBMOD-79	−18.79 ± 4.22
PSMB5_MZBMOD-93	−4.15 ± 2.75
PSMB5_MZBMOD-50	−11.00 ± 3.21
PSMB5_MZBMOD-99	−7.51 ± 3.38

**Table 7:** In silico pharmacokinetics and ADMET properties of Marizomib (MZB) and its top-performing analogs.

Parameter	MZB	MZBMOD-77	MZBMOD-79	MZBMOD-93	MZBMOD-50	MZBMOD-99
Molecular Weight (g/mol)	313.78	481.58	407.50	335.39	359.42	363.45
Hydrogen Bond Acceptors (HBA)	4	6	6	6	5	5
Hydrogen Bond Donors (HBD)	2	2	2	1	1	2
cLogP	0.513	2.810	1.591	1.597	0.716	2.671
Total Surface Area	217.61	354.78	298.60	244.84	245.13	282.73
Polar Surface Area (PSA)	75.63	105.23	101.93	76.07	83.91	92.70
Relative PSA	0.278	0.257	0.280	0.256	0.268	0.261
CYP Inhibitor	None	CYP3A4	CYP3A4	None	None	None
Mutagenic	Low	None	None	High	None	None
Tumorigenic	High	None	None	None	None	None
Reproductive Effective	High	None	None	None	None	None
Irritant	None	Low	None	High	None	High
Shape Index	0.524	0.428	0.413	0.375	0.423	0.577
Molecular Flexibility	0.398	0.323	0.308	0.247	0.405	0.455
Molecular Complexity	0.930	0.963	0.893	0.845	0.948	0.838
Solvent Accessible Surface Area (SASA)	509.102	690.075	579.515	504.734	592.286	631.913
Hydrophobic Component of SASA (FOSA)	262.447	423.787	455.525	340.156	412.699	461.339
Hydrophilic Component of SASA (FISA)	120.387	86.831	94.686	105.606	141.693	156.493
Percent Human Oral Absorption	78.905	100.000	90.684	77.373	78.031	73.843
QPlogHERG	−2.071	−3.252	−1.726	−3.750	−2.556	−2.726
QPPCaco	336.704	1026.878	741.274	246.230	324.836	194.782
QPlogBB	−0.494	−0.397	−0.497	0.079	−0.886	−1.258
QPPMDCK	868.818	759.945	631.349	120.321	208.185	146.808
QPlogKp	−3.168	−2.200	−2.681	−5.225	−3.709	−3.778
QPlogKhsa	−0.867	0.105	−0.211	−0.080	−0.750	−0.525

## Data Availability

The original contributions presented in this study are included in the article/Supplementary Material. Further inquiries can be directed to the corresponding author(s).

## Supplementary Materials

The following supporting information can be downloaded at: https://www.mdpi.com/article/10.3390/ijms262412159/s1.

## Abbreviations

The following abbreviations are used in this manuscript.

ADMET	Absorption, distribution, metabolism, excretion, and toxicity
AIRs	Ambiguous interaction restraints
BBB	Blood–brain barrier
CNS	Central nervous system
CYP	Cytochrome P450
DDI	Drug–drug interaction
DFT	Density functional theory
FEP	Free-energy perturbation
FMO	Frontier molecular orbital
GAFF2	General amber force field
GBM	Glioblastoma
HADDOCK	High ambiguity driven protein-protein docking
HBA	Hydrogen bond acceptor
HBD	Hydrogen bond donor
HOMO	Highest occupied molecular orbital
LBD	Ligand-binding domain
LUMO	Lowest unoccupied molecular orbital
MD	Molecular dynamics
MM/PBSA	Molecular mechanics/Poisson-Boltzmann surface area
MOE	Molecular operating environment
NPT	Number of particles, pressure, and temperature
NVT	Number of particles, volume, and temperature
PME	Particle mesh Ewald
PSMB5	Proteasome subunit beta type-5
PRODIGY	Protein binding energy prediction
RMSD	Root mean square deviation
RMSF	Root mean square fluctuation
RoG	Radius of gyration
SASA	Solvent-accessible surface area
SCF	Self-consistent field
SPCE	Single point charge extended
SPR	Surface plasmon resonance
STP	Single-trajectory protocol
TPSA	Topological polar surface area
UPS	Ubiquitin–proteasome system
